# Burnout and Its Relationship with Demographic and Job-Related Variables among Dentists in Lithuania: A Cross-Sectional Study

**DOI:** 10.3390/ijerph18083968

**Published:** 2021-04-09

**Authors:** Eglė Slabšinskienė, Andrej Gorelik, Aistė Kavaliauskienė, Apolinaras Zaborskis

**Affiliations:** 1 Department of Oral Health and Paediatric Dentistry, Medical Academy, Lithuanian University of Health Sciences, A. Mickevičiaus 9, LT-44307 Kaunas, Lithuania; andrej.gorelik@gmail.com; 2 Department of Orthodontics, Faculty of Odontology, Medical Academy, Lithuanian University of Health Sciences, A. Mickevičiaus 9, LT-44307 Kaunas, Lithuania; aiste.kavaliauskiene@lsmuni.lt; 3 Department of Preventive Medicine & Health Research Institute, Faculty of Public Health, Medical Academy, Lithuanian University of Health Sciences, A. Mickevičiaus 9, LT-44307 Kaunas, Lithuania; apolinaras.zaborskis@lsmuni.lt

**Keywords:** dentist, burnout, prevalence, risk factors, job satisfaction, Maslach Burnout Inventory, Lithuania

## Abstract

Although burnout has been described as a serious hazard for personal and professional lives and has been surveyed among dentists in many countries, no study has been published regarding burnout among dentists in Lithuania. This study aimed to evaluate the burnout level among Lithuanian dentists and its association with demographic variables, job satisfaction, and other job-related variables. The data were collected among dentists online or during professional conferences while using an anonymous questionnaire (*n* = 380). The Maslach Burnout Inventory (MBI) was used to evaluate the burnout level. A Poisson regression was applied for the analysis of relationships between variables. We observed that 42.3% of the respondents had a high emotional exhaustion (EE) (95% confidence interval (CI): 37.4–42.3%), while 18.7% (95% CI: 15.0–22.9%) and 28,2% (95% CI: 23.4–32.6%) had high depersonalization (DP) and low personal accomplishment (PA), respectively. Nonetheless, 15.3% (95% CI: 11.8–18.9%) of the study population experienced a high level of overall burnout. An original job satisfaction index was elaborated. It was significantly associated with sum scores of all burnout dimensions: with the EE sum score (Ratio of Sum Score Means (RSSM) 1.54; 95% CI: 1.46–1.62), DP sum score (RSSM 1.59; 95% CI: 1.45–1.74), and PA sum score (RSSM 0.88; 95% CI: 0.84–0.92). It was concluded that Lithuanian dentists can be characterised by high burnout intensity and high prevalence of burnout, being especially evident in emotional exhaustion. The dentist with low job satisfaction appeared to be the most vulnerable to all burnout dimensions.

## 1. Introduction

Burnout syndrome has been described as a serious personal and professional disorder among professionals in different sectors, as well as in healthcare and medicine [1,2,3]. Professional burnout among dentists has also been examined for many decades [4,5]. The studies provided evidence that dentistry is a stressful profession that is related to working with ever-changing technologies and methods of practice, treating anxious patients, dealing with high physical demands and high expectations from self as well as patients, and having a heavy workload [6,7]. Accumulated stress provokes burnout [8,9,10,11]. Previous studies have shown that dentistry is turning into fatigue-inducing profession [9,12]. In addition, there is evidence that dental burnout can harm patient safety (e.g., imperfect dental infection control) [11,13], and patients are more likely to give low satisfaction ratings when visiting healthcare professionals who suffer from burnout [14]. Therefore, it is not surprising that studies conducted in many countries have assessed the burnout level and the prevalence of high burnout among dental professionals [8,15,16,17,18,19].

The practice of dentistry in Lithuania has undergone substantial changes in recent decades [20]. After the restoration of Lithuania’s independence in 1990, with the establishment of a free market in the country, dentistry was one of the first of health care sectors to turn very rapidly from a public and free-of-charge dental care system to a two-tier dental delivery model, including both private and public dentistry. Commercial aspects started to play a significant role in dental practice. In essence, the dental profession has lost its specific sacred activity that is raised by the physician. Dental practitioner started to get multiple stress, because he had to carry out all of the clinical, administrative, and managerial tasks, while, at the same time, there were strong restrictions, requirements, and control by governmental and sanitary authorities. Consequently, it is important to understand how prevalent burnout is among country’s dentists, and how it is in comparison with research data from other countries.

Maslach and her colleagues [21] have studied the burnout syndrome for many years and invented its measurement tool—the Maslach Burnout Inventory (MBI), which is still considered to be the golden standard for assessing the burnout [22]. A version of this inventory was adopted for human services survey [23]. The authors followed the three-dimension model of burnout [21]. The first dimension is defining emotional exhaustion (EE) as the feelings of not being able to give more of oneself on an emotional level and a decrease of one’s own emotional resources; the second dimension is defining depersonalization (DP), or cynicism, as a negative distance response, cynical feelings, and behaviours towards other people, who usually are the users of the service or care; and, the third dimension is defining a reduced personal accomplishment (PA) as the decrease in one’s own feelings of competence and achievement at work. The dimensions are estimated by summing the scores of the respective MBI subscales. Based on the mean of the sum score, the burnout level, or intensity, in the population is estimated [24]. Although there are no rigorous criteria for assessing high levels of burnout, scientific studies still attempt to present the prevalence of burnout in occupational groups. In light of these limitations, previous literature indicates a burnout prevalence of 8 to 36% among dental professional in European countries [25].

To gain new insight into and increase the understanding of the burnout problem, researchers in different professional sectors are looking for factors that determine, or even are associated with, the level and prevalence of burnout. Numerous systematic reviews and meta-analyses on burnout among health workers have been published worldwide providing evidence that heavy workload, low job satisfaction, and job stressors are all associated with one or more dimensions of burnout, first and foremost with emotional exhaustion [26,27,28,29,30]. The problem of burnout among Lithuanian doctors and nurses has also been investigated [31,32,33]. Still, burnout associates in dentistry has been investigated in a relatively small number of countries and findings on the relationship between burnout and its associates are inconsistent within the literature [8,9,34]. Overall, the current literature implies the need for an extended examination of professional burnout and associated factors among dentists in order to develop effective burnout control measures. So far, this kind of research has not been conducted in Lithuania.

Among the job-related associates, job satisfaction must be taken into consideration. Job satisfaction is an important construct that is known to be associated with workers’ performance and wellbeing [35]. A lack of job satisfaction is expected to be a predictor of burnout, although some studies conclude that there is a bidirectional and longitudinal relationship between burnout and job satisfaction [35,36,37]. Job satisfaction also correlated with work engagement or, conversely, some authors tested work engagement as a predictor of job satisfaction [18,38,39,40,41]. Despite many different psychometric instruments existing to measure the job satisfaction construct, so far few of those instruments have shown satisfactory validity evidence [35]. Indeed, this indicator should be straightforward, but it reflects key aspects of the dentist job satisfaction, such as earnings, psychological climate, relationships with colleagues and the head of the clinic, and, in general, whether such work could be recommended to young people in choosing their profession. The question arises as to whether such a construct would correlate with dental burnout.

The present study has two main objectives. The first is to evaluate the level and prevalence of professional burnout among dental practitioners in Lithuania in order to compare the relevance of the burnout problem in the context of other countries. The second objective is to examine the relationship between burnout level and demographic variables, job satisfaction and other job-related variables in order to increase the understanding of aetiology of the burnout syndrome. In line with these objectives, the first hypothesis was that Lithuanian dentists, as compared to their counterparts in other countries, have a high level of burnout. Secondly, it was hypothesised that certain demographic and job-related characteristics, including job satisfaction, appear to be related to higher burnout levels. The study should provide the analytical framework that is necessary for the design of effective intervention programs to reduce burnout among dentists in Lithuania.

## 2. Materials and Methods

### 2.1. Sample Size Calculation

The software G*Power 3.1 (University of Dusseldorf, Dusseldorf, Germany) [42] was helpful in determining the number of subjects that would provide a reliable estimation of the association between the MBI sum score and individual dentist’s characteristics. Because the MBI sum score was expected to be deviated from the normal distribution, according to Čekanavičius and Murauskas [43] and Hayat and Higgins [44], it was approximated by a Poisson distribution and a corresponding regression method was chosen to assess the relationship between variables. The calculation procedure assumed 80% power, a confidence level of 5%, a mean of sum score 10, binomial predictors, and an effect size to be detected of at least 1.1. The minimum sample size that was required by these parameters was 260. Assuming a response rate of 50%, the sample size was increased to 520. The license to use the MBI among the required number of respondents was purchased from the copyright holder (Mind Garden, Inc., Menlo Park, CA, USA).

### 2.2. Participants and Data Collection

This was an observational study with a cross-sectional design. The survey was conducted in October 2019–February 2020, in two ways. First, the data were collected using hard questionnaire copy among dentists who attended professional conferences in five regions of Lithuania. With the consent of the conference organizers, 400 registered participants were randomly selected (80 attendees at each conference) and, at the registration desk, were invited to participate in the study. Of them, 246 dentists completed the questionnaire; the response rate was 61.5%. These respondents can be considered to be a randomized subsample. As the number of subjects was still insufficient, data collection was continued by online survey in closed Facebook groups “Odontologijos profesionalai” (“Professionals of Odontology”) and “Lietuvos odontologai” (“Dentists of Lithuania”). Facebook readers were asked not to complete this questionnaire again if they had already completed this questionnaire during the conference. In this way, 150 completed questionnaires (all purchased online copies) were received; however, the response rate for this group of respondents remained unknown and it was a non-randomized subsample as its participant pool may not include every dentist of the country. Altogether, in both subsamples, 396 dentists participated in the study. A comparison of these subsamples according to demographic and other factors did not show statistically significant differences, so it was decided to analyse the whole sample. Because 16 respondents did not answer all MBI questions they were excluded; therefore, the final study sample comprised of 380 dentists.

The data collection conformed to the principles that were outlined in the World Medical Association’s Declaration of Helsinki. It was approved by the Bioethics Committee Center of the Lithuanian University of Health Sciences on 16 October 2019 (permission No. BEC–OF–13). The anonymity and confidentiality of the participants was guaranteed. At all times, the data were processed according to Lithuanian data protection laws.

### 2.3. Measures

We adopted the MBI-HSS (Maslach Burnout Inventory—Human Services Survey) to evaluate levels of burnout among dentists [23]. The instrument was translated into Lithuanian and it was previously validated in other fields of medicine [32,33]. In recent study, we have conducted the factorial validation of MBI among Lithuanian dentists [45]. The inventory comprised 22 items with responses being rated on a seven-point Likert scale ranging from 0 (“never”) to 6 (“daily”). The MBI included three subscales (dimensions): emotional exhaustion (EE; nine items with sum score range of 0 to 54); depersonalization (DP; five items with sum score range of 0 to 30); and, personal accomplishment (PA; eight items with sum score range of 0 to 48) [23].

Information regarding the participants was also collected; it included age, gender, and other demographic characteristics. Because this study was focused on job-related issues, the questionnaire included questions on work practice experience, type of practice, working places, specialist status, work duration, etc. The origins of these questions came from their logical connection with burnout or from the current situation of dentistry in Lithuania, or they have been formed based on literature sources (e.g., an item on the specialization of the dentist was formed according to the study of Zini et al. [46]). An additional set of five questions was assigned to determine job satisfaction. These questions were inspired by other studies that measured job satisfaction [35,47]. The questions were related to salary satisfaction, how much the co-workers and the boss value the employee, how the psychological climate of the workplace is assessed, and, finally, whether the respondent would recommend young people to choose the profession of dentist. For the purposes of the present analysis, positive answers to these questions were coded “+1”, negative “−1”, especially negative “−2”, and neutral “0” (see Results). The total score was a job satisfaction index. Its values could vary from −6 to +5. Because respondents who were the heads of the dental clinic could not answer the question about the employee’s relationship with the head, their index could range from −5 to +4. This difference in index metrics required that some of the statistics be recalculated in subsample of respondent without clinic heads (*n* = 330).

### 2.4. Statistical Analysis

The mean sum score for each MBI dimension (EE, DP, and PA) was calculated and treated as continuous variable. In addition, an average item rating (mean sum score/number of items) and mean adjusted to maximum sum score (mean/maximum sum score × 100%) were computed. A category of burnout level was assessed following manual guidelines [48]. Consistent with previous similar studies [8,15,33], the subjects were categorized into one of the three groups—a high, moderate, or low level of burnout. Those with EE sum score of ≥27 were considered to have high EE level, 17–26 moderate, and 0–16 low level. Those with DP sum score of ≥13 were considered to have high DP level, 7–12 moderate and 0–6 low level. Those with PA score of ≤31 were considered to have low PA level, 32–38 moderate, and ≥39 high level. The subjects with high EE, high DP, and low PA levels were simultaneously considered to have a high level of overall burnout. Others were considered to have low overall burnout. We estimated the crude rates of burnout levels (low, moderate, and high) and 95% confidence intervals (CI) of these results. A Pearson χ^2^ test of association to describe the relationship between categorical variables and Spearmen’s rho pairwise correlation coefficient to describe the relationship between continuous variables were employed. The Cochran–Armitage test (statistic *T_CA_*) was used to assess the linear trend of a high burnout level prevalence by job satisfaction score.

A detailed analysis of the burnout relationship with the demographic and work-related variables was conducted using generalized linear models with Poisson distribution and log link function [43,44,49]. The sum score of any of the burnout dimensions was considered to be a continuous dependent (outcome) variable in the Poisson regression; meanwhile, the individual dentist’s characteristics were considered to be independent predictors. The strength of the relationship between the sum score and predictor was assessed by the ratio of sum score means (RSSM), which shows how many times the mean of the outcome variable (sum score) increases as the predictor changes from the reference category to the contrast category. The model fit to the existing data was assessed by deviance/degree of freedom; its values being close to 1 indicated a good model fit. In addition, the likelihood ratio *χ*^2^ in omnibus test with *p* < 0.05 showed that several independent variables were significantly related to the dependent variable. The model was fitted and run separately for each domain (EE, DP, and PA) using univariate analysis with one independent variable and multivariate analysis with all sets of independent variables.

All of the analyses were conducted at a *p* < 0.05 level of significance using SPSS statistical package (version 21.0; SPSS Inc., Chicago, IL, USA; 2012). We followed the Strengthening the Reporting of Observational Studies in Epidemiology (STROBE) cross-sectional checklist when writing this article [50].

## 3. Results

### 3.1. Demographic and Job-Related Characteristics

Table 1 provides the profile of demographic and job-related characteristics of the dentists who participated in the study. The study population comprised of 380 dentists, 15.3% males and 84.7% females. The participants ranged in age from 23 to 80 years, with a mean age of 37.3 (SD 12.9) years, 33.7% of the dentists were 40 or more aged. More than half of the participants were married (59.3%) and had children (52.6%). Approximately 46% of the dentists have professional experience 10 or more years. By specialty, 73.7% of the study participants were general dental practitioners and 26.3% were specialists that had acquired postgraduate qualification (e.g., in paediatric dentistry, oral surgery, orthodontics, etc.). The average workload per week was 36.7 h (SD 12.2). There was also a variety of subjects according to the other job-related characteristics of interest (number of working places, type of practice, property of clinics).

### 3.2. Job Satisfaction

Table 2 shows the distribution of dentists’ responses to questions regarding job satisfaction. The feeling of stress in the workplace microclimate was the main factor diminishing job satisfaction; there were 52.9% of the dentists who claimed more or less this feeling. Approximately one in five (21.2%) respondents complained about a low salary. Almost the same proportion (18.7%) of respondents reported about a head of clinic who did not appreciate their efforts. A relatively small group of dentists (11.8%) could not assess the latter issue because they were the managers of the clinic themselves. Accordingly, every third respondent (32.9%) would not recommend young people to choose the profession of dentist. Summarizing all of the responses about job satisfaction, a sum score was calculated. Table 3 presents the frequency of job satisfaction scores in the study sample. As predicted, the scores among the ordinary dentists ranged from −6 to +5, whereas, among the heads of dental clinic, ranged from −5 to +4. Because of the small number of respondents, groups with job satisfaction scores of −6 and −5 were merged in future analysis. It was found that 28.8% of dentists have obtained a negative score and 59.7% of dentists have obtained a positive score of job satisfaction (11.5% of respondents had a score of zero). The prevalence of negative and positive job satisfaction values did not differ significantly between subsamples of the ordinary dentists and heads of dental clinic. This finding indicates that an aggregated measure of job satisfaction (negative and positive scores of job satisfaction) can be used in total sample, regardless of difference in index metrics. The prevalence of negative and positive job satisfaction scores also did not differ significantly between males and females, as well as between younger (<40 years) and older (≥40 years) respondents (Table S1).

### 3.3. Maslach Burnout Inventory and Level of Burnout

Table 4 shows the statistical and psychometric characteristics of the MBI sum score by burnout dimensions. These data indicate that respondents were likely to report higher scores for items of the EE dimension when compared with items of the PD while the distribution of responses to the PA items was like a mirror image of the distribution of other dimensions. The Kolmogorov–Smirnov’s test for normality of the distribution of sum score was not significant for the EE dimension, but not for the PD and PA dimensions. Assessments of Cronbach’s alpha ranged from 0.737 for DP dimension to 0.904 for EE dimension, indicating from an acceptable to good internal consistency reliability of the MBI dimensions (this assessment for the whole inventory was 0.896).

**Figure 1:**
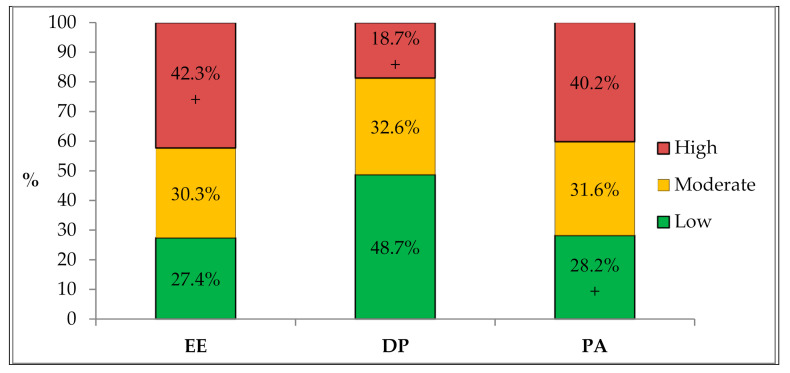
Sum score levels by MBI dimensions. Abbreviations: EE—emotional exhaustion; DP—depersonalization; PA—personal accomplishment. “+” indicates proportions assigned to high burnout.

Figure 1 shows the burnout levels by MBI dimensions. Approximately 42% of the respondents had a high EE level (42.3%; 95% CI: 37.4–42.3), while 18.7% (95% CI: 15.0–22.9) and 28.2% (95% CI: 23.4–32.6) had a high DP and low PA level, respectively. Nonetheless, 15.3% (95% CI: 11.8–18.9) of respondents had a high level of overall burnout (i.e., high EE, high DP, and low PA).

### 3.4. Maslach Burnout Inventory and Job Satisfaction

**Figure 2:**
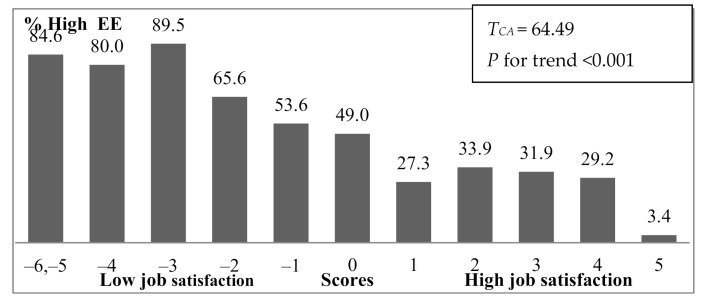

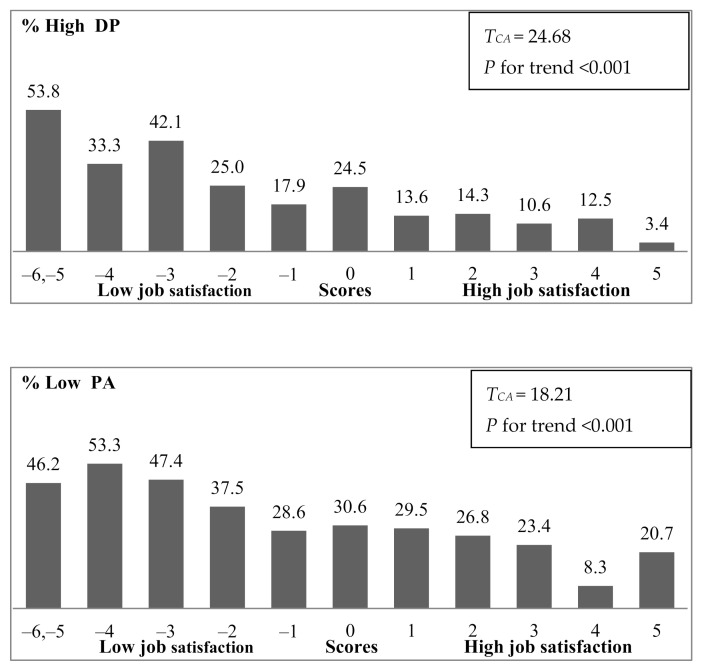
Percentage of high emotional exhaustion (EE), high depersonalization (DP), and low personal accomplishment, by job satisfaction scores. The respondents with a job satisfaction score of −6 or −5 were pooled into one group. *T_CA—_*Cochran-Armitage statistic.

In total sample, the job satisfaction score positively correlated with the PA sum score (rho = 0.292; *p* < 0.01) and negatively correlated with the EE sum score (rho = −0.520; *p* < 0.01) and DP sum score (rho = −0.342; *p* < 0.01). These data indicate that those with higher job satisfaction had a lower sum score of the EE and DP dimensions, and a higher sum score of the PA dimension; consequently, a lower risk for high burnout of each dimension. Figure 2 illustrates trends of percentages of high EE (*T_CA_* = 64.49; *p* < 0.001) and DP (*T_CA_* = 24.68; *p* < 0.001), and low PA (*T_CA_* = 18.21; *p* < 0.001), by job satisfaction scores (in subsample of the ordinary dentists, Cochran–Armitage statistics *T_AC_* were 58.06, 16.97, and 14.49, respectively, for all of them *p* < 0.001). The prime trend of these percentages by job satisfaction scores was found for the high EE, which was in line with the results of correlation analysis.

### 3.5. Relationship of Burnout Level with Demographic and Job-Related Variables

The relationships between the MBI dimension sum score, job satisfaction level, and a group of 11 demographic and workload variables were analysed by Poisson regression. Only significant relationships from a multivariate analysis are shown in Table 5 (the results from a univariate analysis can be find in the Supplementary Table S2). Although, in the univariate analysis, the EE sum score was related to all independent variables, except the number of working days per week, in the multivariate analysis, only five significant (*p* < 0.05) independent variables were found: work practice experience 10 or more years, and postgraduate dental specialization decreased the EE sum score while being female, working more than 40 h per week, and low job satisfaction, especially, increased the EE sum score. With regard to the DP sum score, four significant independent variables were selected: dentists of older age (40 or more years aged), and specialists’ groups had a lowered mean sum score, and, similarly to EE, dentists working more than 40 h per week, and having low job satisfaction, especially, were characterised by an increased mean DP sum score. Personal accomplishment that was assessed by its mean sum score was significantly diminished among never married, working less than five days per week, and dentists stood out with decreased job satisfaction.

## 4. Discussion

The results of our study indicate that approximately one of seven dentists (15.3%) of Lithuania suffers from pronounced burnout. According to the selected criteria, high emotional exhaustion was the most common (42.3%), but other high dimensions of burnout—depersonalization and personal accomplishment—also occurred frequently. All of the burnout dimensions were significantly associated with job satisfaction. A number of demographic variables (gender, age, and marital status) and job-related variables (work practice experience duration, specialist status, numbers of clinics and their property in the practice, number of hours and days per week spent in practice) were also identified as being associated with some aspects of the burnout syndrome. The prevalence of burnout and factors that are associated with burnout in dentists have been thoroughly investigated [51]. However, the direct comparison of our findings with the published literature across different countries must be interpreted with caution as the prevalence and magnitude of associations may vary among the studies with different methods and criteria of burnout definition [22].

Most of the research has used the Maslach Burnout Inventory with three dimensions (MBI) [21,23]. It has been estimated that the MBI is becoming a golden standard for burnout assessment [22]. Despite its popularity, this tool is still criticized. Recently, Schaufeli et al. (2020) have identified that MBI suffers from its conceptual, psychometric, and practical shortcomings; therefore, they developed a new Burnout Assessment Tool as an alternative to the MBI [52]. Although the new tool is more complex than MBI (it has 33 items divided into six subscales), it is already used in some studies [53].

Much debate exists regarding where to establish cut-off points and whether continuous or dichotomous measures should be used [24,54]. The third edition of the MBI Manual [48] proposed dividing the sample into three groups for each subscale. Later, the authors of the inventory state that burnout should be conceptualised as a continuous variable, rather than as a dichotomous variable that is either present or absent [23,55,56]. Therefore, for the comparison of data between studies, it is appropriate to take the intensity of burnout measured by means or medians of continuous sum score of its dimensions into account. However, many authors still for practical purposes seek to estimate the prevalence of burnout using several criteria for its categorisation into a risk group. According Rotenstein et al. [22], the most frequent definition of overall burnout, used by 17.2% (21/122) of studies, required individuals to score all of at least 27, at least 10, and no more than 33 on the MBI exhaustion, depersonalization, and personal accomplishment subscales, respectively. Regarding occupational burnout, according to Maslach et al. [48], the degree of burnout is high if the EE, DP, and PA sum scores are ≥27, ≥13, and ≤31, respectively. A similar definition of overall high burnout has also been adopted in our study to allow for comparability with other studies.

The studies conducted among dental professionals in UK (2008) [15], the US (2017) [18], and Hong Kong (2017) [8] used the same criteria for the assessment of burnout prevalence that we used. The percentage distribution of overall burnout of the current study (7.9%) was similar with that reported in UK (8.0%) and Hong Kong (7.0%), and almost twice lower than in the US (13.2%). In addition to the already mentioned studies, in the literature we found more studies (in Brazil (2013) [19], Korea (2015) [16], and Taiwan (2019) [11]), which were conducted in populations of dentist using the same burnout examination methodology, but only data on the intensity of burnout dimensions were available. When comparing these data, we found that the greatest variation was across the means of the EE score. The magnitude of the EE estimate in the current study (24.3) differed slightly from that of the UK (25.1), Korea (24.2), and Taiwan (26.3), but it was markedly higher than in the surveys among the US (18.0), Hong Kong (19.4), and Brazil (19.5) dentists. There was low heterogeneity in the intensity of the DP and PA dimensions of burnout when comparing data across countries. The proportion (15.3%) of Lithuanian dentists experiencing significant burnout in all three dimensions (overall prevalence) was the highest among dentists within the selected countries (Hong Kong (2017) 7.0% [8]; the UK (2008) 8.0% [15]; and, the US (2017) 13.2% [18]). The mean scores of EE and DP were higher and the mean score of PA was lower, as well as overall prevalence of burnout was higher when compared to other medical staff in Lithuania whereas the same methodology was applied [33]. These comparisons suggest that Lithuanian dentists can be characterised by high burnout intensity and high prevalence of burnout syndrome, especially evident in emotional exhaustion. These results are possibly due to different work environments, dental care systems, social support, and occupational safety supervision policies.

The literature reviews state a lack of studies that explore the causation of burnout in dentistry [25,51]. Therefore, in further analysis, we examined the relationships between the burnout level and demographic variables, job satisfaction, and other job-related variables. For this analysis, a Poisson regression was employed as the most appropriate method [43]. This choice was due to several reasons. First, not all MBI dimensions had a sum score that satisfied normality of distribution. Second, the relationship between variables could be more precisely assessed if several variables were taken into account, so the model had to be multivariate. Third, and the most import reason, the literature states that burnout is best considered to be a continuum, rather than a dichotomous variable, as of now, the diagnostic criteria have not been well-specified and the necessary clinical research has not been done [23,55,56]. Indeed, the best analytic approach is to evaluate the relationship between symptoms of burnout and outcomes while using a sum score of individual domains as continuous data.

According to Singh et al. [51], the levels of the burnout dimensions tend to vary in relation to the gender. The findings of our study were in line with this conclusion indicating that female dentists were more likely than male dentists to report higher scores of the EE dimension. Likewise, Maslach and Jackson [21] suggested that females are more prone to emotional exhaustion than males. In contrast, other studies found that male suffer from burnout more than females because they have a higher workload than females [16,51]. For DP and PA dimensions, a significant difference between genders was not detected, presumably because the proportion of males in the sample was much smaller than that of females. Thus, this problem has limited more detailed insight into the role of gender in the risk of burnout.

There was some variation in burnout level by age and years of work practice. In our study, younger dentists (up to 40 years) had a significantly higher DP mean than older ones (40 and more years), but a significant difference between age groups was not observed for EE and PA dimensions. Our findings confirm the results of earlier studies, which showed that burnout most often occurs in the first years of a professional career [16,21,46,51]. Accordingly, the dentists in the older age range of our sample may be those who have already survived the early professional stress within the first few years of career and, at present, they do well in their professional activity. These findings coexist with variation in the EE dimension by years of work experience, which was revealed in this study and reported in previous studies [12,46], regardless of the contrary data [17].

There was also variation in the burnout level by marital status, but it was only manifested in the PA dimension in such a way that newer married dentists were more likely to rate poorer personal accomplishment. In the literature, several authors [57,58] suggested that married people would be more resistant to the burnout because they feel familial responsibility with greater ability to face emotional problems, while other authors [19,59] identified no relationship between burnout manifestation and marital status.

Variations in burnout dimensions were also related to workload variables. The sum scores of the EE and DP dimensions were significantly associated with dentists having full working week (>40 h per week). This fact deals directly with increased working hours, which, according to many authors, increase the risk for burnout anxiety and loneliness [17,46,47]. However, it is surprising that working days per week positively affected the personal accomplishment of dentists. This fact is evident from the increased mean of sum score of the corresponding burnout dimension.

General dentists are the majority of the dental workforce; however, according to the findings from our study, they were more prone than specialist dentists (oral surgeons, prosthodontists, orthodontists, or endodontists) to report higher burnout scores in the EE and DP dimensions. Although studies on the burnout syndrome among dental specialists are sparse, it was seen that dentists with more competent skills and knowledge were less stressed, and thus had a lower burnout risk [17,38,46,60]. Therefore, encouragement and support of dental specialty promotion programs may contribute to lessen professional burnout among dentists [46,60,61].

Job satisfaction is another important factor to be observed. Based on the well-known fact that satisfaction with clinical work is determined by factors, such as earnings, psychological climate, relationships with colleagues and the head of the clinic, and, in general, whether such work could be recommended to young people in choosing their profession, for the purposes of the present study we proposed a simple five-item tool to assess “job satisfaction”. Applying the positive and negative weights to answer categories, a sum score (index) was calculated; its negative values led to less job satisfaction, and the positive values led to higher job satisfaction. In our study, the proposed job satisfaction index was associated with all three dimensions of burnout. The dentist with low job satisfaction (i.e., with negative index values) appeared to have higher sum score mean of the EE and DP dimensions and a lower sum score mean of the PA dimension as compared with the dentists with high job satisfaction (i.e., with positive index values). In our study, the values of relationship between job satisfaction and burnout level were as strong and significant as were found in other similar studies on burnout and job satisfaction [37,40,41,62].

### 4.1. Strengths

To our knowledge, this is the first study to report the findings on burnout level and prevalence among Lithuanian dentists after the recent validation of the MBI in this professional group [34]. This study also sought to examine the association between burnout and several factors of dentist work. One strength of the study is that the original job satisfaction factor was constructed and its significance for burnout risk was assessed. Another strength of the study is a Poisson regression analysis used to assess the relationship between burnout and associated factors. In statistics [43], it is the most reliable method for describing the relationship between a Poisson distributed response variable (a count) and one or more independent variables (predictors). However, such a method has never been used in a burnout study of this kind.

### 4.2. Limitations

This study has a few limitations. First, a limitation of our study is its cross-sectional nature. It is known that, in cross-sectional studies, causal relationships could not be established since the data on risk factors and outcomes are assessed at the same time [63]. Significant associations found in this study between burnout dimensions and other factors could be fully explained in longitudinal studies [29]; however, so far there is a lack of such studies in dentistry [51]. Nevertheless, a cross-sectional study is important for its usefulness in the search for risk factors and in the planning of healthcare policies.

Second, the survey was based on participants who voluntarily provided personal information, which may be biased. On the one hand, those respondents who were more prone to answer the questionnaire were also more likely to overestimate burnout symptoms. On the other hand, the respondents who had high level of burnout might felt the questions to be too sensitive and, thus, have been unwilling to participate in survey [8]. Unfortunately, the level of burnout remained unknown among non-participants.

Third, we used an original tool for measuring job satisfaction. However, this measure had one limitation, because its metrics (range of the measured values) depended on the status of the respondent, i.e., whether he was an ordinary dentist or, at the same time, a head of the dental clinic. Additional calculations in the subsample of ordinary dentists revealed that this limitation was not essential. Notwithstanding this result, the validity of the proposed measure requires further investigation, but this objective is outside the scope of the current article.

Despite these limitations, we believe that current findings show the issues that need special attention to decrease the level and prevalence of professional burnout among Lithuanian dentists and, thus, improve all of the dental care system as well as increase well-being of dentists.

## 5. Conclusions

Lithuanian dentists can be characterised by a high burnout intensity and high prevalence of burnout. This is especially evident in the dimension of emotional exhaustion (mean score was 24.3; prevalence of EE score ≥27 was 42.3%). The dentist with low job satisfaction appeared to be most vulnerable to all burnout dimensions, while other job-related variables and demographic characteristics had an ambiguous association with burnout dimensions. The findings of the present study can be used in the planning of strategies for burnout prevention and intervention programs among dentists in Lithuania.

## Figures and Tables

**Table 1:** Distribution of study participants by demographic and workload characteristics.

Characteristics	No. (%) or Mean (SD) of Respondents	
Gender (*n* = 380)		
Male	58	(15.3)
Female	322	(84.7)
Age (years) (*n* = 374)		
Mean and standard deviation	37.3	(12.9)
< 40	248	(66.3)
≥ 40	126	(33.7)
Marital status (*n* = 376)		
Married	223	(59.3)
Never married	119	(31.6)
Divorced / widow	34	(9.0)
Number of children (*n* = 377)		
No children	177	(46.9)
Have children	200	(52.6)
Work practice experience (*n* = 365)		
Mean and standard deviation	12.8	(12.3)
<10	196	(53.7)
≥10	169	(46.3)
Working in several places (*n* = 372)		
In one clinic only	183	(49.2)
In two or more clinics	189	(50.8)
Type of practice (*n* = 378)		
Public	59	(15.6)
Private	213	(56.3)
Both	106	(28.1)
Property of the clinic (for private practice only) (*n* = 329)		
Commercial network	79	(24.0)
Individual (small) clinics)	250	(76.0)
Specialist status (*n* = 380)		
General dental practitioners	278	(73.7)
Specialists	102	(26.3)
Number of working hours per week (*n* = 371)		
Mean and standard deviation	36.7	(12.2)
≤40	257	(69.3)
>40	114	(30.7)
Number of working days per week (*n* = 376)		
<5	83	(21.8)
≥5	293	(77.1)

**Table 2:** Dentists’ responses to questions about job satisfaction and scores given to assess overall job satisfaction.

Characteristics	No. (%) of Respondents		Scores Given
Do you think you are earning enough given your workload and efforts? (*n* = 378)			
Really enough	113	(29.9)	+1
O.K., though I would like to earn more	185	(48.9)	0
My earning is insufficient / low	80	(21.2)	−1
Do you feel sufficiently respected and valued by your colleagues in the clinic or in a professional group? (*n* = 378)			
Yes	274	(72.5)	+1
No	39	(10.3)	−1
I never thought about it	65	(17.2)	0
Do you feel sufficiently respected and appreciated by the head of clinic? (*n* = 374)			
Yes	209	(55.9)	+1
No	70	(18.7)	−1
I never thought about it	51	(13.6)	0
I myself am the head of the clinic	44	(11.8)	0
How do you assess the microclimate in your workplace? (*n* = 376)			
I like the atmosphere at work, I don’t feel any stress about it	167	(44.4)	+1
The atmosphere at work is quite normal, but of the atmosphere at work I sometimes feel stress	174	(46.3)	−1
I don’t like the atmosphere at work because it causes a lot of stress	25	(6.6)	−2
The atmosphere at work does not affect my mood and well-being	10	(2.7)	0
Would you recommend the profession of dentist to an undecided young person? (*n* = 377)			
Yes	131	(34.7)	+1
No	124	(32.9)	−1
I can’t reply	122	(32.4)	0

**Table 3:** Frequency of job satisfaction scores in studied sample.

No. of Scores	No. (%) of Respondents					
	Total Sample (*n* = 374)		Ordinary Dentists (*n* = 330)		Heads of the Dental Clinic (*n* = 44)	
−6	3	(0.8)	3	(0.9)	-	
−5	10	(2.7)	8	(2.4)	2	(4.5)
−4	15	(4.0)	15	(4.5)	0	(0.0)
−3	19	(5.1)	16	(4.8)	3	(6.8)
−2	32	(8.6)	30	(9.1)	2	(4.5)
−1	28	(7.5)	22	(6.7)	6	(13.6
0	46	(12.3)	40	(12.1)	6	(13.6)
+1	43	(11.5)	37	(11.2)	6	(13.6)
+2	55	(14.7)	47	(14.2)	8	(18.2)
+3	46	(12.7)	40	(12.1)	6	(13.6)
+4	48	(12.8)	43	(13.0)	5	(11.4)
+5	29	(7.8)	29	(8.8)	-	
<0	107	(28.6)	94	(28.5)	13	(29.5)
0	46	(12.3)	40	(12.1)	6	(13.6)
>0	221	(59.1)	196	(59.4)	25	(56.8)
			χ^2^ = 0.13, df = 2, *p* = 0.936			

**Table 4:** Statistical and psychometric characteristics of the Maslach Burnout Inventory dimensions (*n* = 380).

Dimension	Number of Items	Sum Score					Cronbach’s Alpha
		Mean	Standard Deviation	Average Item Rating ^a^	Mean adjusted to Maximum Sum Score (%) ^b^	Test for Normality ^c^	
Emotional exhaustion	9	24.27	11.66	2.70	44.94	0.163	0.904
Depersonalization	5	7.78	5.94	1.56	25.93	<0.001	0.737
Personal achievement	8	35.56	7.66	4.45	74.08	0.011	0.789

Note: ^a^ Mean/Number of items; ^b^ Mean/Maximum sum score × 100%; ^c^ Kolmogorov-Smirnov test.

**Table 5:** Relationship between emotional exhaustion, depersonalization and personal accomplishment levels, and the significant independent variables: results from multivariate Poisson regression analysis ^a^.

Independent Variable	Emotional Exhaustion			Depersonalization			Personal Accomplishment		
	RSSM	95% CI	*p* Value	RSSM	95% CI	*p* Value	RSSM	95% CI	*p* Value
Gender									
Females	1.11	1.04–1.19	0.001						
Males *	1.00								
Age (years)									
≥40				0.74	0.65–0.84	<0.001			
<40 *				1.00					
Marital status									
Divorced/widow							0.98	0.92–1.06	0.640
Never married							0.95	0.90–0.99	0.017
Married *							1.00		
Work practice experience (years)									
≥10	0.87	0.81–0.93	<0.001						
<10 *	1.00								
Specialist status									
Specialists	0.91	0.86–0.97	0.003	0.87	0.78–0.96	0.007			
General dental practitioners *	1.00			1.00					
Number of working hours per week									
>40	1.09	1.04–1.15	0.001	1.11	1.01–1.21	0.026			
≤40 *	1.00			1.00					
Number of working days per week									
<5							0.92	0.88–0.97	0.001
≥5 *							1.00		
Job satisfaction score									
Negative	1.61	1.53–1.69	<0.001	1.67	1.54–1.82	<0.001	0.88	0.84–0.92	<0.001
0	1.31	1.23–1.40	<0.001	1.24	1.10–1.40	<0.001	0.97	0.92-1.03	0.300
Positive *	1.00			1.00			1.00		

Notes: ^a^ Only significant relationships are presented; * Reference group; RSSM—Ratio of sum score means.

## Data Availability

The dataset analyzed during the current study is not publicly available due to the restrictions of the local ethics committee and institutional data security and privacy policies. The data access request needs institutional and ethics committee’s approval.

## Supplementary Materials

The following are available online at https://www.mdpi.com/article/10.3390/ijerph18083968/s1, Table S1. Frequency (%) of negative and positive values of job satisfaction score, by gender and age. Table S2. Relationship between emotional exhaustion, depersonalization and personal accomplishment levels, and the significant dependent variables: results from univariate Poisson regression analysis.

## Abbreviations

CI	Confidence interval
DP	Depersonalization
EE	Emotional exhaustion
MBI	Maslach Burnout Inventory
PA	Personal accomplishment
RSSM	Ration of sum score means
SD	Standard deviation
