# Supplementary material for: Burnout and Its Relationship with Demographic and Job-Related Variables among Dentists in Lithuania: A Cross-Sectional Study
Source: Int J Environ Res Public Health. 2021 Apr 9;18(8):3968. doi: 10.3390/ijerph18083968 (PMC8069627; doi:10.3390/ijerph18083968)
Supplement: Supplementary file 1 [file ijerph-18-03968-s001.pdf]

Table S1. Frequency (%) of negative and positive values of job satisfaction score, by gender and age.

	Job satisfaction score			*P* value*
	**Negative**	**0**	**Positive**	
Gender				
Males	12 (21.4)	7 (12.5)	37 (66.1)	0.421
Females	95 (29.9)	39 (12.3)	184 (57.9)	
Age (years)				
< 40	70 (28.5)	29 (11.8)	147 (59.8)	0.848
≥ 40	35 (28.5)	17 (13.8)	71 (57.7)	
Total	107 (28.6)	46 (12.3)	221 (59.1)	

Note: * χ^2 test.

Table S2. Relationship between emotional exhaustion, depersonalization and personal accomplishment levels, and the significant independent variables: results from univariate Poisson regression analysis.

Independent variable	Emotional exhaustion			Depersonalization			Personal accomplishment		
	RSSM	95% CI	*P* value	RSSM	95% CI	*P* value	RSSM	95% CI	*P* value
Gender									
Females	1.16	1.10–1.24	<0.001	1.09	0.99–1.21	0.088	0.99	0.94–1.04	0.621
Males*	1.00			1.00			1.00		
Age (years)									
≥ 40	0.88	0.85–0.93	<0.001	0.73	0.68–0.80	<0.001	1.00	0.97–1.04	0.869
< 40*	1.00			1.00			1.00		
Marital status									
Divorced/widow	0.95	0.88–1.03	0.229	0.79	0.68–0.91	0.001	1.00	0.94–1.06	0.990
Never married	1.15	1.10–1.20	<0.001	1.15	1.06–1.24	0.001	0.95	0.91–0.98	0.003
Married*	1.00			1.00			1.00		
Number of children									
Have children	0.86	0.83–0.89	<0.001	0.81	0.76–0.87	<0.001	1.03	1.00–1.07	0.063
No children*	1.00			1.00			1.00		
Work practice experience (years)									
≥ 10	0.84	0.83–0.89	<0.001	0.75	0.70–0.81	<0.001	1.03	1.00–1.07	0.097
< 10*	1.00			1.00			1.00		
Working in several places									
In two or more clinics	1.06	1.02–1.11	0.002	1.05	0.97–1.13	0.210	1.01	0.98–1.05	0.489
In one clinic only*	1.00			1.00			1.00		
Type of practice									
Public	0.95	0.90–1.01	0.121	0.90	0.81–1.00	0.049	0.97	0.94–1.01	0.176
Public and private	1.08	1.03–1.14	0.001	1.07	0.99–1.16	0.095	0.99	0.95–1.04	0.736
Private*	1.00			1.00			1.00		
Property of the clinic									
Individual clinic	0.94	0.90–0.98	0.021	0.87	0.80–0.95	0.002	0.99	0.95-1.03	0.495
Commercial network*	1.00			1.00			1.00		
Specialist status									
Specialists	0.81	0.77–0.85	<0.001	0.77	0.70–0.84	<0.001	1.06	1.02–1.10	0.005
General dental practitioners*	1.00			1.00			1.00		
Number of working hours per week									

> 40	1.20	1.15–1.25	<0.001	1.26	1.17–1.36	<0.001	0.98	0.95–1.02	0.981
≤ 40*	1.00			1.00			1.00		
Number of working days per week									
< 5	0.98	0.93–1.03	0.433	0.88	0.80–0.96	0.005	0.95	0.91–0.99	0.019
≥ 5*	1.00			1.00			1.00		
Job satisfaction score									
Negative	1.68	1.60–1.75	<0.001	1.76	1.63–1.91	<0.001	0.88	0.85–0.92	<0.001
0	1.34	1.26–1.42	<0.001	1.30	1.17–1.46	<0.001	0.95	0.91–1.01	0.073
Positive*	1.00			1.00			1.00		

Notes: * Reference group; RSSM – Ratio of sum score means.
